# New York Pathological Society

**Published:** 1879-11

**Authors:** 


### NEW YORK PATHOLOGICAL SOCIETY.

New York, September 24, 1879.

*Subperiosteal Excision of Elbow.*—Dr Lange exhibited a patient upon whom he had performed subperiosteal excision of the right elbow-joint, after Prof. Voight's method, and gave the following history: Patient was nineteen years of age, and had a stiff right elbow from his early childhood. He knew nothing about the cause of this trouble. The arm remained thin and powerless, and any exercising caused pain in the joint, especially during the last two years. Various methods of treatment were employed but without avail, and the patient finally resolved to get rid of his trouble by an operation.

At that time the patient was in good health. The right elbow was anchylosed at an angle of 90°, with a mobility of nearly 10°. The tissues about the joint were somewhat thickened, but no fistula or other indication of existing suppuration was present. The olecranon and head of the radius seemed thickened, and they were very painful on pressure, showing chronic ostitis. The operation was performed on the 25th of June by means of a bilateral incision, antiseptically made without spray, and by the bloodless method. The periosteum was carefully presesved; in all those places where important tendons or ligaments had their insertion (olecranon, coronoid process, epicondyles of humerus, etc.), thin layers of bone were separated by means of hammer and chisel, and remained in connection with the periosteum, according to the plan of Voight.

The operation was somewhat tedious and difficult, the periosteum being thickened and tense, and all recesses of the joint obliterated. Lister's gauze dressing was applied. The after-treatment consisted of a dorsal splint of plaster-of-Paris, with elevated position and slight extension. The position of the arm was about 150°. After the tenth day position was changed every second day to the extent of from 75° to 150°. At the end of the third week, articulated silicate dressings (with shoulder-piece), which had a movable joint and rubber strips, were applied corresponding to the new joint. The joint allowed a slipping of the bones of the forearm upward and backward, according to the physiological position of the ulna. Active and passive movement were freely practiced by causing the patient to lift a box filled with sand, the amount of which was increased every day. These exercises effected a stretching of the elbow. The arm, by means of the strip, was held in a right angle. The weight of the sand was chosen always a little beyond the strength of the patient to master it, so that it slowly extended the arm, the patient endeavoring to prevent this, and struggling against the weight by the power of his muscles. The rubber strips kept up a passive dragging of the ligaments and held the bones in a certain adaptation.

After the seventh week the apparatus could be omitted, the new formation of the bones being very significant, and almost perfect cicatrization had been complete at the end of the fifth week. There had not been any significant discharge since the second week, only those places discharging superficially where the drainage-tube had been introduced.

The reaction after the excision had been quite insignificant. A bloody infiltration of the arm and forearm disappeared under the physiological changes of color, and was reabsorbed without interfering with the healing process.

When the patient was exhibited to the Society, just three months after the operation, the elbow presented nearly its normal shape. Motion was between 80 and almost 180 degrees, without any abnormal lateral movability. The condyles of humerus appeared stronger than normal. Pro- and supination almost normal (had been exercised also methodically every day, the apparatus being removed for this purpose·) The head of the radius was well marked, and normally faced the external condyle. The olecranon was distinctly formed, but was a little smaller than normal. Above its apex something like a sesamoid bone could be felt in the triceps tendon. The arm was so strong that the patient was able to lift a chair, seizing it by the leg, and after stretching the arm he held the chair a good while in the air. The flexion to an acute angle was difficult. The specimen showed deep depressions in the articular part of the ulna, especially one behind the base of the coronoid process. Its walls were hard and smooth; they were covered by a dense fibrous tissue which surrounded a small quantity of cheesy matter. All the depressions in the bone were filled with a succulent fibrous tissue, which sent vascularized adhesions to the opposite cartilage; so the process was on its way to cicatrization. The bones of the humerus were almost normal; cartilage of radius showed some cicatricial depressions; its head had an abnormal process toward the articulation with the ulna, which was entirely obliterated by a dense fibrous mass.

*Erect Luxation of the Humerus.*—A vigorous laborer, about forty-five years of age, when rolling a heavy piece of ice along an inclined plane, lost his balance, and, in tumbling forward, seized, by the elevated right hand, the projecting border of a barrel of beer, a series of them forming a comparatively narrow path. He fell down in spite of that, and felt "that something was turned out in his shoulder when he was in the air." He could not get up alone, and the arm remained in the erect position. Dr. L. saw him eight hours after injury, in consultation with Dr. Assenheimer. The patient was in bed, with the arm elevated to about 120 degrees, stretched in the elbow, hand in supination. He held with his hand a string, which had been attached to the upper border of his bedstead. Every sinking down of the arm caused a severe pain about the upper end of the middle third of the humerus, perhaps in consequence of dragging of the coraco-brachialis muscle; deltoid relaxed. There was a deep depression below the acromion. The head of the humerus could clearly be seen below the coracoid process and exceeding its line toward the middle line so that about the tubercle corresponded to the end of the coracoid process. The axis of the humerus corresponded to a line which might have crossed the articulation of sternum and third rib. If the patient made a strong lateral curvature of the trunk, so that the short ribs almost touched the crest of the ileum, and if at the same time he lowered his scapula as much as possible, he, when sitting, was able to rest the wrist upon the knee. The arm was prevented from falling by the coracoid process, and perhaps the pectoralis minor. The reduction was made without chloroform, patient sitting on a chair. The scapula was fixed by means of a towel.

The elevation was increased, and after extending as much as possible, the arm was lowered, when the head of humerus slipped forward under the coracoid process, and was placed against the border of the glenoid cavity. A further slight rotation inward made it slip to its normal position without difficulty. Recovery took place in a comparatively short time, so that after three weeks he was able to resume his work.

*Large Mammary Tumor.*—Dr. A. C. Post exhibited the segement of an enormous tumor of the mammary gland, which he had removed by operation from a lady over sixty years of age. The whole tumor, after its removal, weighed nineteen pounds. It had existed for about fifteen years, was small when first observed, and grew very slowly. During the past three years it had grown rapidly, and finally reached such a size as to occasion extreme inconvenience and to prey upon her health. Dr. Post saw her early last spring and advised the removal of the growth, at the same time telling the patient that the operation was a hazardous one. She did not make up her mind to follow the advice until last July, when a hemorrhage occurred from an ulceration on the surface of the growth.

The operation was performed with the assistance of Dr. Sands, of this city, and Drs. Barker and Owen, of Morristown, N. J. The subcutaneous vessels were enormously enlarged, and it required very careful dissection to separate the integument from the tumor. The patient became very much exhausted while on the table, requiring repeated hypodermic injections of brandy. After the operation she had a very feeble pulse, and died in the course of an hour. The tumor was fibro-cystic in character.

*Yellow Fever—Fatty Liver—Parenchymatous Change in the Kidneys—Uræmic Coma.*—Dr. Satterthwaite presented the liver, spleen, and kidneys, and microscopic sections from a patient who had died in the Presbyterian Hospital, of yellow fever, on July 29th.

The following was the outline of the clinical history prepared by Dr. Wetmore, of the hospital staff, and Dr. W. H. Porter had prepared the microscopic sections:

Margaret Cregan; æt. 32, Ireland; widow; stewardess; was admitted July 25, 1879. Family history excellent. Personal health had always been good. Her illness dated back only five days, *i. e.*, it commenced July 20, 1879. She then had frontal headache and general "soreness all over her back and legs." At 10 A.M., on the 20th, she had a severe chill, followed by some fever. There was nausea, but no vomiting; there was also some diarrhœa. She had chills night and day, with fever and sweating, for the five days preceding admission. When admitted her temperature was 105° F., pulse 108. The diagnosis of remittent fever was made, and she was given cinchonidia sulph., gr. xx., in divided doses. The symptoms at that time were manifestly insufficient to establish the subsequent diagnosis.

On the following day the pulse had fallen to 98; temperature 104° F.; and in the evening to 92; temperature 104¼° F. The patient slept most of the day. She had some diarrhœa, but no pain. Her diet was milk chiefly. The urine examination gave the following results: sp. gr. 1020; color, amber; reaction, alkaline; odor, ammoniacal; sediment, yellowish-white; albumen, half volume. The amount of urine could not be ascertained, because it was passed with the evacuations from the bowels. Microscopic examinations revealed triple phosphate and urate of ammonia crystals, with some granular casts and blood-corpuscles.

On the seventh day (July 27th) the pulse had again fallen in the morning from the figures of the preceding morning; it was 94; temperature 102¼°. In the evening the pulse was 90, and temperature lower again, (101°).

On July 28th, the eighth day, she vomited occasionally, though the cinchonidia was kept down half an hour. Pulse 88, and temperature the same as the preceding night, *i. e.*, 101 deg. The vomited matter was white (probably the milk she drank.) The diarrhœa continued. The color of the passages was light yellow. Menstruation occurred this afternoon.

July 29th (ninth day).—The night was passed with comparative comfort. Twice she vomited some dark matter. Jaundice was now noticed for the first time. The pulse was now weak and she was delirious, recognizing no one. The second sound of the heart was almost imperceptible. Respiration labored, and 30 per minute. At noon it was found that she had passed no water since 6 A. M. Hot cloths were then applied to the bowels, and she was cupped over the kidneys: no œdema anywhere. Shortly afterward a catheter was introduced, and brought away a drachm of dark urine. It contained 50 per cent. per volume of albumen. The microscope also detected blood and the epithelium that is described as renal. Pulse was 88, and temperature 99$\frac{4}{5}$°.

As it was plain she was suffering from suppression of urine, cups were again applied over the kidneys, and one-quarter of a grain pilocarpin hydrochlor. was injected hypodermically. She died at 1.50 P. M. Autopsical examination twenty-one hours after death, by Dr. W. H. Porter, curator to the hospital, in the presence of Drs. E. C. Janeway, W. De F. Day, and E. B. Ramsdell:

*External Examination.*—Rigor mortis well marked; body well provided with fat, and in a good state of preservation; general jaundice of the skin; numerous ante-mortem ecchymoses on the lower extremities, together with the usual post mortem discolorations on the under surface of the body and limbs.

Dr. Putnam-Jacobi asked if there were any evidences of the re-absorption of bile. This seemed a possible condition in view of the fact that the liver-cells were atrophied.

Dr. Satterthwaite remarked that the liver-cells were filled with oil-globules and granular fat, and were rather swollen than otherwise.

Dr. Putnam-Jacobi thought that a distinction should be made between fatty degeneration in which mere globules were deposited and that condition in which only granules were present.

Dr. Satterthwaite did not think that there was, practically speaking, any difference between these conditions.

Dr. Putnam-Jacobi was of the opinion that a distinction should be made between fatty infiltration and fatty degeneration of liver-cells. In the former cases the cells became broken down, and in the latter their functions were not markedly impaired.

Dr. Satterthwaite thought that the particular size of the oil-globules was not of so much account as their relative position in the substance of the hepatic lobule. If the deposit had extended to the centre of the acinus around the hepatic vein, the function of the liver was interfered with, if not entirely abolished. On the other hand, when the so-called simple fatty degeneration took place, there was usually a deposit of oil merely in the cells at the periphery of the lobule.

### FOREIGN BODY IN THE EYE.

Dr. Knapp exhibited an eye removed by operation from a man sixty years of age. The organ had contained a foreign body for two years. The patient, at the time of the accident, was passing by a man who was pecking a mill stone, when he felt that some sharp body entered his eye. Dr. Knapp saw him at the end of eight months afterwards, and discovered a shrunken and movable cataract in the injured eye. As no foreign body was discovered, Dr. Knapp thought that it was enveloped in the cataract. Last June the patient presented himself again, with well-marked irritation in the eye. Extirpation was advised accordingly. On examining the eye afterward, the membranes, except the crystalline lens, were completely normal. At the bottom of the ciliary processes a small piece of iron was discovered, but there was no new formation of connective tissue. In the absence of the latter, Dr. Knapp did not think it impossible that the foreign body had been originally encapsuled in the cataract, and that by a shrinking of the cataract it had fallen to the position where found.

### AN INTERESTING CASE OF EXOPHTHALMUS.

Dr. Seguin presented a photograph of a patient with a remarkable exophthalmus. The patient came to the Manhattan Eye and Ear Department on account of projecting eyeballs. Dr. Webster found, in connection with that condition, a pulse ranging from 92 to 96, and the anterior portion of the neck normal. There were no other symptoms in the case. The point of interest centred in the fact that the exophthalmus was the only symptom of Grave's disease which was present. Again, the cause of the disease could not be explained by the mere contraction of Muller's muscle. The unusual point in this case was disease of the optic nerve. There were no evidences of a pulsating tumor.

Dr. Knapp referred, in this connection, to sixty cases of exophthalmus which were on record. Only in a very few of these was aneurism found. In quite a large number the carotid was tied, and some of these cases were cured. He had never met with a case of Grave's disease combined with disease of the optic nerve.

Dr. Seguin asked if there was any bruit in these cases.

Dr. Knapp answered that such a bruit was subjective in the majority of cases.

OBLITERATION OF THE PANCREATIC DUCT.

Dr. Beverley Robinson presented a specimen of obliteration of the pancreatic duct due to carcinoma, and accompanied with great emaciation. The history of the case was given in detail:

George Davis, æt. 27 years; English; groom; unmarried. Admitted to hospital June 28, 1879. About one and a half months ago he lost appetite, and about a month ago began to vomit everything he ate soon after taking food. Never saw any blood in vomits. This condition continued up to the present time. Had pain in the stomach after eating. Abdomen has been distended for about two weeks; legs and feet became swollen about the same time. Passes very little water; bowels regular. Has grown weak and thin. Urine normal. Fluid in peritoneal cavity. Liver cannot be made out. Apex-beat of heart in third intercostal space, above the nipple, and synchronous retraction in fourth interspace.

July 30th.—Paracentesis performed, and 344 ounces of pale yellow fluid, somewhat cloudy, was drawn off.

July 31st.—Urine normal. Liver not felt. A nodulated growth was felt in the median line, below the border of the ribs and extending to the right.

Aust 8th.—Scanty urine for several days. To-day passed ℥ ix.

September 18th.—Patient has vomited almost daily since admission. No blood has been seen in the vomit. Died of asthenia.

September 19th.—Post-mortem examination. Rigor mortis well marked. Cadaver much emaciated. Abdominal cavity filled with clear straw-colored fluid, distending the abdominal walls, and pressing up the diaphragm. Lungs and heart very small. Kidneys normal. Intestines atrophied and walls thickened; surface of rectum covered in spots with fibrinous nodules. Mesentery also covered with fibrin. A small piece of omentum only remained floating about loose in the abdominal cavity. Stomach, intestines, pancreas, and liver were firmly adherent. At pyloric end of stomach, on posterior surface, was a tumor involving the pyloric orifice, which was about the size of a goose-quill. The pancreas was almost completely obliterated by the tumor. Liver small. Gall-bladder small, and cystic-duct obliterated; hepatic duct and common bile-duct free.

The question having arisen as to the significance of fat in the stools in diseases of the pancreas, Dr. Putnam-Jacobi remarked that while fatty stools were not always present in such cases, they did sometimes exist when there was simple ulceration of the duodenum.

Dr. Satterthwaite stated that primary cancer of the pancreas was almost unknown. It was almost without exception a secondary disease to cancer of the stomach.

Dr. John C. Peters, in this connection, referred to a case of cancer of the head of the pancreas which came under his observation some months since, in which pressure upon the pylorus caused enormous distention of the stomach. The history and specimen had been presented to the Society.

*Rapid Lithotrity.*—Dr. Keyes exhibited calcuii removed by rapid lithotrity from a physician aged seventy-three. He had suffered from the symptoms of stone for a number of years. The calcui, which weighed altogether ten and a half drachms, were removed in an hour and twenty-five minutes. This long time was due to the difficulty in grasping the largest stone, which was an inch and a half in diameter, and situated in a sacculated portion of the bladder posteriorly.

Dr. Keyes also took occasion to exhibit Sir Henry Thompson's new instrument, which had been used on that occasion.

Dr. Keyes also exhibited small renal calculi which had evidently passed through the ureter into the bladder without producing pain.

Dr. Peters referred to a similar case in his practice.—*Medical Record.*

